# 6% Hydroxyethyl starch (HES 130/0.4) diminishes glycocalyx degradation and decreases vascular permeability during systemic and pulmonary inflammation in mice

**DOI:** 10.1186/s13054-017-1846-3

**Published:** 2018-05-01

**Authors:** Andreas Margraf, Jan M. Herter, Katharina Kühne, Anika Stadtmann, Thomas Ermert, Manuel Wenk, Melanie Meersch, Hugo Van Aken, Alexander Zarbock, Jan Rossaint

**Affiliations:** Department of Anesthesiology, Intensive Care and Pain Medicine, University Hospital of Münster, Albert-Schweitzer-Campus 1, Building A1, 48149 Münster, Germany

**Keywords:** Hydroxyethyl starch 130/0.4, Inflammation, Vascular permeability, Glycocalyx, Sepsis

## Abstract

**Background:**

Increased vascular permeability is a pathophysiological hallmark of sepsis and results in increased transcapillary leakage of plasma fluid, hypovolemia, and interstitial edema formation. 6% hydroxyethyl starch (HES 130/0.4) is commonly used to treat hypovolemia to maintain adequate organ perfusion and oxygen delivery. The present study was designed to investigate the effects of 6% HES 130/0.4 on glycocalyx integrity and vascular permeability in lipopolysaccharide (LPS)-induced pulmonary inflammation and systemic inflammation in mice.

**Methods:**

6% HES 130/0.4 or a balanced electrolyte solution (20 ml/kg) was administered intravenously 1 h after cecal ligation and puncture (CLP) or LPS inhalation. Sham-treated animals receiving 6% HES 130/0.4 or the electrolyte solution served as controls. The thickness of the endovascular glycocalyx was visualized by intravital microscopy in lung (LPS inhalation model) or cremaster muscle (CLP model). Syndecan-1, hyaluronic acid, and heparanase levels were measured in blood samples. Vascular permeability in the lungs, liver, kidney, and brain was measured by Evans blue extravasation.

**Results:**

Both CLP induction and LPS inhalation resulted in increased vascular permeability in the lung, liver, kidney, and brain. 6% HES 130/0.4 infusion led to significantly reduced plasma levels of syndecan-1, heparanase, and hyaluronic acid, which was accompanied by a preservation of the glycocalyx thickness in postcapillary venules of the cremaster (0.78 ± 0.09 μm vs. 1.39 ± 0.10 μm) and lung capillaries (0.81 ± 0.09 μm vs. 1.49 ± 0.12 μm).

**Conclusions:**

These data suggest that 6% HES 130/0.4 exerts protective effects on glycocalyx integrity and attenuates the increase of vascular permeability during systemic inflammation.

**Electronic supplementary material:**

The online version of this article (doi: 10.1186/s13054-017-1846-3) contains supplementary material, which is available to authorized users.

## Background

Sepsis is among the leading causes of death among hospitalized patients, and epidemiologic data indicate an incidence of 200–1000 cases per 100,000 inhabitants in populations in the USA and Sweden [1, 2]. The underlying causes are manifold, but the common pathophysiological course includes excessive overactivation of the immune system and increased microvascular permeability [3]. This may result in increased transcapillary leakage of plasma fluid, hypovolemia, and interstitial edema [4, 5]. Hypovolemia leads to decreased cardiac output, resulting in reduced systemic oxygen delivery and generalized vasoconstriction, which may eventually impair organ function. Correction of low plasma volume therefore may be essential to maintaining adequate organ perfusion and oxygen delivery. If untreated, organ failure and eventually death may be the consequence [6].

The clinical treatment of patients with sepsis and hypovolemia requires the use of both vasopressors to maintain an adequate vascular smooth muscle tonus and intravascular fluid administration. Resuscitation fluids differ in their molecular composition and can roughly be classified in crystalloid and colloid solutions. For decades, there has been a debate regarding the use of crystalloids or colloids, as well as regarding the efficacy of different colloids. In contrast to colloids, crystalloids have small effects on coagulation; there is no risk of inducing allergic reactions; and they are inexpensive [7]. Crystalloids are relatively ineffective, however, as plasma volume expanders because their osmotic particles pass freely across the capillary membrane, resulting in rapid distribution to the extracellular space, thus diminishing the hemodynamically relevant volume that remains intravascular. Consequently, relatively large volumes must be infused to restore intravascular normovolemia, raising an adverse risk of tissue edema. However, colloid solutions remain in the bloodstream for a longer time because of their composition and structure [8]. The oncotic effects of colloids may also reinforce their plasma-expanding capacity. Nonetheless, the plasma-expanding effect of colloids is transient because of continuous clearance from the circulation related to the rate of degradation and renal loss as well as continuous leakage of macromolecules into the interstitial space. According to the modern two-pore theory of transvascular exchange, transcapillary leakage of macromolecules occurs through the large pores of the capillary/venular membrane and also appears to be greater at increased permeability [9]. In addition to the size and number of the large pores, transcapillary leakage may be influenced by the charge of the molecules and their interaction with the glycocalyx and other endothelial structures [10]. However, current research indicates that the (extended) use of colloid solutions is associated with increased frequency of renal replacement therapy in critically ill patients [11]. As a consequence, a black box warning regarding the use of colloids in these patients has been issued by the U.S. Food and Drug Administration and the European Medicines Agency.

The endothelial glycocalyx is a dynamic structure localized at the luminal side of the endothelium. It plays a central role in the context of vascular permeability because it functions as a barrier between blood plasma and the endothelium [12]. The main components of the glycocalyx are membrane-bound proteoglycans and glycoproteins incorporating plasma- and endothelium-derived soluble components [13]. The glycocalyx actively fulfills important physiological functions by signal sensing and by transmission to the endothelial surface and shielding it from access by cellular components in the bloodstream [14]. Sepsis leads to degradation of the endothelial glycocalyx, which is related to altered vascular permeability [15]. Glycocalyx degradation is accompanied by the release of its soluble components into the bloodstream (e.g., syndecan-1 and hyaluronan [hyaluronic acid]), a process that is actively mediated by cleavage enzymes, including heparanase and hyaluronidase [16]. Several strategies aimed at protecting the glycocalyx from degradation or repair after degradation have been proposed [17–19]. However, no clinical therapy for glycocalyx protection or repair has yet succeeded.

To date, there have been no studies comparing the effects of contemporary colloid solutions and crystalloids on the distribution of fluids, the integrity of the glycocalyx, and vascular permeability specifically under noninflammatory and inflammatory conditions. The present study was designed to evaluate the distribution of 6% hydroxyethyl starch HES 130/0.4 (Volulyte®; Fresenius Kabi, Bad Homburg, Germany) and a balanced crystalloid electrolyte solution (Isolyte®; Fresenius Kabi) under baseline and inflammatory conditions, as well as the effects of these solutions on the integrity of the glycocalyx and vascular permeability in murine models of pulmonary and systemic inflammation.

## Methods

### Animals and reagents

We used 8–12-week-old C57BL/6 mice. The mice were kept in a barrier facility under specific pathogen-free conditions. All animal experiments were approved by the Landesamt für Natur, Umwelt und Verbraucherschutz Nordrhein-Westfalen, Germany (animal protocols 84-02.04.2012.A373 and 84-02.04.2011.A377) and were carried out in accordance with the National Institutes of Health Guide for the Care and Use of Laboratory Animals. Unless otherwise stated, all reagents were obtained from Sigma-Aldrich (Taufkirchen, Germany).

### Murine models of systemic and pulmonary inflammation

Systemic inflammation was induced by cecal ligation and puncture (CLP) [20]. Mice were anesthetized by intraperitoneal injection of ketamine (125 μg/g body weight; Pfizer, New York, NY, USA) and xylazine (12.5 μg/g body weight; Bayer, Leverkusen, Germany). Sufficient anesthesia for the analgesic tolerance of surgical procedures was ensured by monitoring the response to a toe-pinch. The animals were killed by exsanguination through transcutaneous cardiac puncture after the experiment. CLP was performed as described before [20]. Briefly, a midline laparotomy incision was made after skin disinfection. The cecum was ligated 14 mm distal to the ileocecal valve so that continuity was preserved, and then it was punctured twice with a 20-gauge needle. It was returned to the peritoneal cavity, and the wound was closed in two layers. Mice were then allowed to recover and had free access to food and water. Animals in the sham group underwent the identical procedure without CLP. One hour after the procedure, the animals were administered 20 ml/kg HES 130/0.4 (Volulyte®) or a control solution (Isolyte® balanced electrolyte solution) by intravenous injection into the tail vein through an intravascular catheter. Volulyte® and Isolyte® share the same electrolyte constituency (Na^+^ 137 mmol/L, K^+^ 4 mmol/L, Mg^2+^ 1.5 mmol/L, Cl^−^ 110 mmol/L, CH_3_COO^−^ 34 mmol/L, osmolality 286.5 mmol/L). The animals were randomized to treatment groups, and the investigators were blinded to these groups. An animal group size of eight mice was analyzed for each condition. Pulmonary inflammation induced by inhalation of lipopolysaccharide (LPS) from *Salmonella enteritidis* was performed as described previously [21]. Evans blue (EB) extravasation as a measure of vascular permeability was analyzed as described previously [22]. EB is a dye that binds to serum albumin after intravenous injection [23]. To investigate the influence of HES 130/0.4 administration on vascular permeability, mice were subjected to a sham or CLP operation and received a dose of 20 ml/kg HES 130/0.4 or Isolyte® (as a control) 1 h after surgery. EB (dissolved in 0.9% saline) was injected at a dose of 20 mg/kg body weight 30 minutes before the animals were killed. After the animals were killed, blood was obtained and centrifuged, and plasma was preserved for EB measurements. Afterward, the animals were subjected to perfusion through the right ventricle with PBS. The organs were harvested and weighed, and the tissue was homogenized in PBS (organ weight in milligrams × 3.5 = PBS volume in microliters) by mechanical mincing using a tissue homogenizer and incubated with formamide (vol/vol 2:1 ratio). The mixture was incubated for 12–18 h at 60 °C. After the incubation, the tissue homogenate was centrifuged at 5000 rpm for 30 minutes. EB absorbance measurements of the supernatants were performed on a spectrophotometer at 620 nm and 740 nm. The maximum absorption of EB occurs at 620 nm. No absorption occurs at 740 nm; the absorption at this wavelength is indicative of the amount of contaminating heme pigments. The degree of leak of EB is represented as a ratio of the corrected absorbance of EB in the tissue to the corrected absorbance of EB in the plasma. The extravasation of EB into peripheral organs was analyzed 4, 8, and 24 h after surgery. All animals survived the 24-h observation period. Each animal was examined at only one time point (4 h, 8 h, or 24 h), and we investigated eight mice at each time point for each experimental group in both models.

### Intravital microscopy of the cremaster and the lung

To investigate the influence of HES 130/0.4 administration on glycocalyx integrity during systemic inflammation in vivo, we performed intravital microscopy (IVM) of the cremaster muscle and lung as described previously [24–27]. Following induction of general anesthesia, the cremaster muscle was exteriorized and prepared for imaging on a custom-built stage. IVM was carried out on an upright microscope (Axioskop; Carl Zeiss, Göttingen, Germany) with × 40 magnification and a 0.75 numerical aperture saline immersion objective. For IVM of the lung, a right lateral thoracotomy was performed, and the lung was positioned under the window of a custom-built fixation device. A mild vacuum was applied to hold the lung in position during microscopy. Images were captured with a charge-coupled device camera (Sensicam; PCO, Kehlheim, Germany). Fluorescein isothiocyanate (FITC)-dextran (150 kDa) was used to assess the glycocalyx width in these experiments as previously described (*see also* the scheme in Fig. 3a) [28–30]. HES 130/0.4 or Isolyte® (20 ml/kg) was administered 1 h after CLP or sham surgery. IVM was performed after 4, 8, or 24 h. A different animal was used at each time point (4, 8, or 24 h) for IVM because it was not possible to continuously monitor a single animal over the whole observation period. Analysis of the recorded data files was performed by blinding the type of fluid used before evaluation.

### Quantification of syndecan-1, heparanase, hyaluronic acid, and hyaluronidase plasma levels and hyaluronidase activity

Plasma levels of syndecan-1, heparanase, hyaluronic acid, and hyaluronidase were quantified using enzyme-linked immunosorbent assay (ELISA) kits following the manufacturers’ instructions (syndecan-1 and heparanase, Cusabio Biotech, College Park, MD, USA; hyaluronic acid, BlueGene, Shanghai, China; hyaluronidase, USCN Life Science, Wuhan, China). Hyaluronidase activity was analyzed using an ELISA-based assay (Echelon Biosciences, Salt Lake City, UT, USA).

### Statistics

Statistical analysis was performed using IBM SPSS version 22.0 software (IBM, Armonk, NY, USA) with the Wilcoxon test or the *t* test as appropriate. More than two groups were compared using two-way analysis of variance followed by the Bonferroni test. Data distribution was assessed using the Kolmogorov-Smirnov test or the Shapiro-Wilk test. All data are presented as mean ± SEM. A *p* value < 0.05 was considered statistically significant.

## Results

### HES 130/0.4 affects plasma levels of different markers of glycocalyx integrity during systemic and pulmonary inflammation

**Fig. 1:**
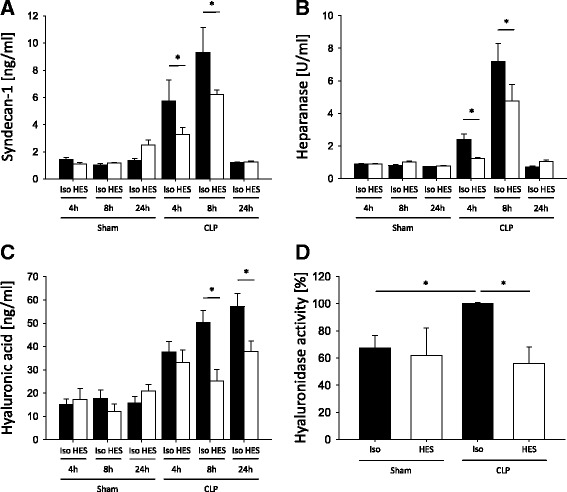
Hydroxyethyl starch (HES) 130/0.4 effects on syndecan-1, heparanase, hyaluronic acid plasma levels, and hyaluronidase activity during systemic inflammation. Wild-type mice underwent a sham or cecal ligation and puncture (CLP) operation (*n* = 8). Sixty minutes after the procedure, mice received 20 ml/kg Isolyte® (Iso) or HES 130/0.4 (HES) as an infusion over 1 h. The plasma levels of (**a**) syndecan-1, (**b**) heparanase, and (**c**) hyaluronic acid were analyzed 4, 8, and 24 h after the operation. **d** Serum hyaluronidase enzyme activity was analyzed 24 h after the CLP procedure and normalized to the CLP Iso group. Data are presented as mean ± SEM, * *p* < 0.05

**Fig. 2:**
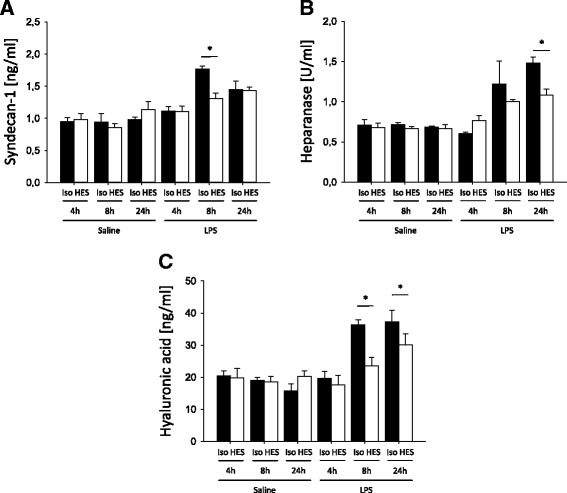
Hydroxyethyl starch (HES) 130/0.4 effects on syndecan-1, heparanase, and hyaluronic acid plasma levels during pulmonary inflammation. Wild-type mice were exposed to nebulized saline or lipopolysaccharide (LPS) (*n* = 8). Sixty minutes after the procedure, mice received 20 ml/kg Isolyte® (Iso) or HES 130/0.4 (HES) as an infusion over 1 h. The plasma levels of (**a**) syndecan-1, (**b**) heparanase, and (**c**) hyaluronic acid were analyzed 4, 8, and 24 h after the procedure. Data are presented as mean ± SEM, * *p* < 0.05

To investigate the integrity of the glycocalyx under different inflammatory conditions, we determined the plasma levels of syndecan-1, hyaluronic acid, and heparanase. After induction of systemic inflammation, syndecan-1, heparanase, and hyaluronic acid levels in the plasma increased over the first 24 h (Fig. 1a–c). Four and eight hours following CLP, the plasma levels of syndecan-1 and heparanase were significantly reduced in HES-treated mice compared with Isolyte®-treated mice (Fig. 1a and b). Mice in the HES-treated groups also had significantly reduced hyaluronic acid plasma levels 8 and 24 h following CLP compared with the Isolyte® control groups (Fig. 1c). In contrast, hyaluronidase plasma levels showed a trend toward higher levels in the HES 130/0.4 group at 8 and 24 h after CLP, which did not reach statistical significance (Additional file 1: Figure S1). To specifically address the higher, although statistically not significant, hyaluronidase levels in CLP animals following HES administration, we performed an additional hyaluronidase enzyme activity assay. Interestingly, we could detect a statistically significant reduction in hyaluronidase activity in blood samples from CLP animals that had received HES compared with Isolyte®-treated animals (Fig. 1d). This could possibly explain why HES administration reduces circulating hyaluronic acid concentrations, whereas hyaluronidase serum concentrations appear to be increased, although this did not reach statistical significance in the CLP model. We also analyzed the plasma levels of syndecan-1, hyaluronic acid, hyaluronidase, and heparanase in mice after LPS-induced pulmonary inflammation (Fig. 2). HES 130/0.4 significantly decreased the plasma levels of syndecan-1 after 8 h, but not after 24 h, compared with the Isolyte® control group (Fig. 2a). Furthermore, HES 130/0.4 significantly decreased the plasma levels of heparanase and hyaluronic acid plasma levels 8 and 24 h after LPS exposure (Fig. 2b and c).

### HES 130/0.4 attenuates glycocalyx degradation in postcapillary venules of cremaster muscle after CLP induction

**Fig. 3:**
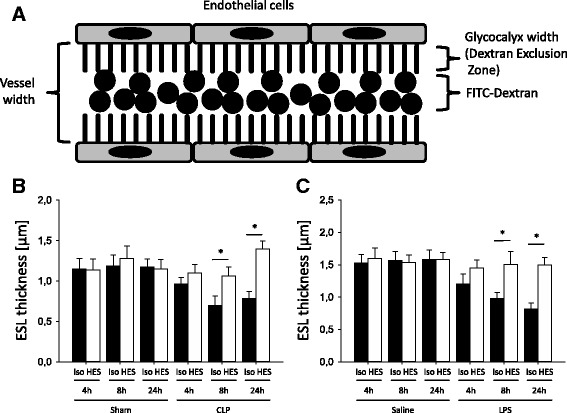
Hydroxyethyl starch (HES) 130/0.4 effects on the thickness of the glycocalyx during systemic and pulmonary inflammation. **a** Schematic illustration of the fluorescein isothiocyanate (FITC) exclusion technique used to visualize the glycocalyx (ESL = endothelial surface layer) width in the cremaster and pulmonary microcirculation in vivo. Mice underwent cecal ligation and puncture (CLP) or a sham operation (**b**) or lipopolysaccharide (LPS) or saline inhalation (**c**), which was 1 h later followed by an infusion of 20 ml/kg Isolyte® (Iso) or HES 130/0.4 (HES) over a period of 1 h. At 4, 8, and 24 h after the operation or inhalation, respectively, the thickness of the glycocalyx was measured in the cremaster muscle in the CLP experiment and in the lung capillaries in the LPS inhalation experiment by intravital microscopy using the FITC-dextran exclusion technique. Data are presented as mean ± SEM, * *p* < 0.05

Systemic inflammation, as induced, for example, by CLP, causes degradation of the vascular glycocalyx [28]. The width of the vascular glycocalyx, analyzed using a FITC-dextran exclusion technique (Fig. 3a), remained unchanged in animals that received Isolyte® or HES 130/0.4 after sham surgery at all time points (Fig. 3b). The induction of systemic inflammation following CLP caused a significant decrease in glycocalyx thickness in animals that received Isolyte® compared with sham animals after 8 and 24 h (Fig. 3b). HES 130/0.4 administration significantly attenuated glycocalyx degradation after 8 h (0.70 ± 0.12 μm vs. 1.06 ± 0.11 μm) and 24 h (0.78 ± 0.09 μm vs. 1.39 ± 0.10 μm) compared with Isolyte®-treated mice, with no significant differences within the HES-treated mice (Fig. 3b). To investigate whether HES 130/0.4 also affects glycocalyx degradation during inflammatory processes in the lung, mice were exposed to nebulized LPS or saline, and HES 130/0.4 or Isolyte® (20 ml/kg) was administered after 1 h. Saline inhalation did not cause glycocalyx degradation, and control mice that received Isolyte® or HES 130/0.4 after saline inhalation did not show significant differences in glycocalyx thickness (Fig. 3c). The induction of pulmonary inflammation by LPS inhalation caused a significant decrease in glycocalyx thickness after 8 and 24 h in animals that received Isolyte® compared with control animals (Fig. 3c). HES 130/0.4 administration significantly attenuated glycocalyx degradation after 8 h (0.98 ± 0.09 μm vs. 1.50 ± 0.20 μm) and 24 h (0.81 ± 0.09 μm vs. 1.49 ± 0.12 μm) (Fig. 3c).

### HES 130/0.4 reduces vascular permeability during systemic and pulmonary inflammation

**Fig. 4:**
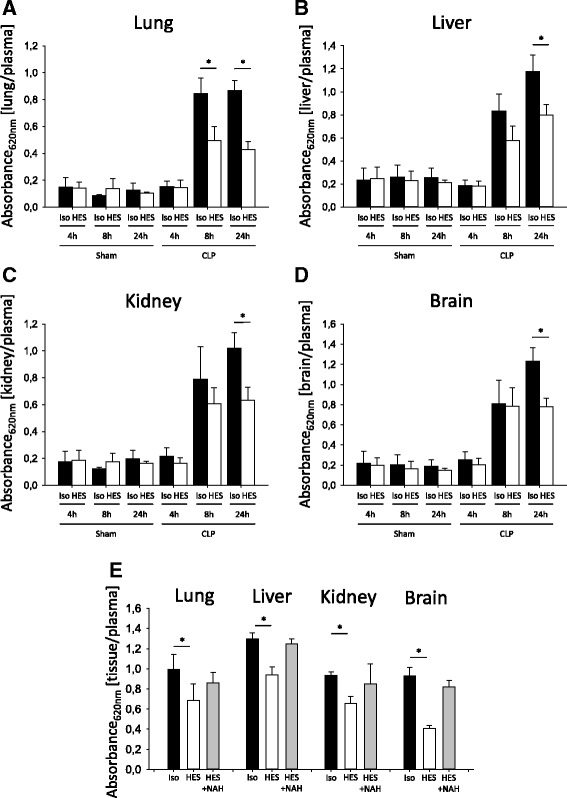
Hydroxyethyl starch (HES) 130/0.4 effects on vascular permeability during systemic inflammation. Wild-type mice underwent a sham or cecal ligation and puncture (CLP) operation (*n* = 8). Sixty minutes after the procedure, mice received 20 ml/kg Isolyte® (Iso) or HES 130/0.4 (HES) as an infusion over 1 h. At 4, 8, and 24 h after the operation, vascular permeability in the (**a**) lung, (**b**) liver, (**c**) kidney, and (**d**) brain was measured by photometry using the extravasation of Evans blue (EB) technique. (**e**) EB extravasation in CLP mice treated with 20 ml/kg Isolyte® (Iso), HES 130/0.4 (HES), or HES 130/0.4 plus 150 μg of *N*-desulfated/re-*N*-acetylated heparin (NAH) 24 h after CLP induction. Data are presented as mean ± SEM, * *p* < 0.05

**Fig. 5:**
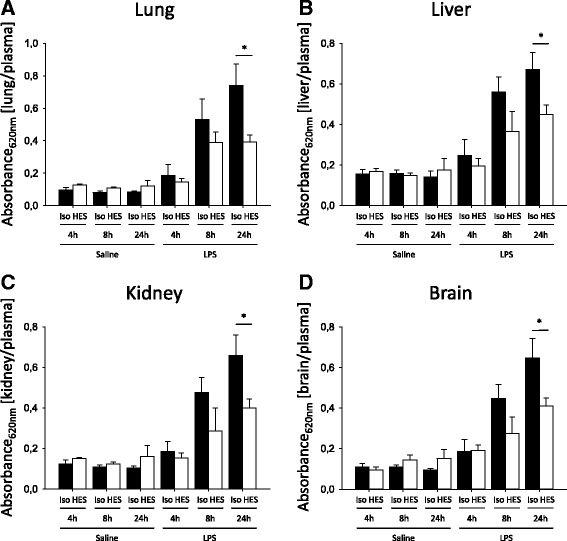
Hydroxyethyl starch (HES) 130/0.4 effects on the vascular permeability during pulmonary inflammation. Wild-type mice were exposed to nebulized saline or lipopolysaccharide (LPS) (*n* = 8). Sixty minutes after the procedure, mice received 20 ml/kg Isolyte® (Iso) or HES 130/0.4 (HES) as an infusion over 1 h. At 4, 8, and 24 h after the operation, vascular permeability in the (**a**) lung, (**b**) liver, (**c**) kidney, and (**d**) brain was measured by photometry using the extravasation of Evans blue technique. Data are presented as mean ± SEM, * *p* < 0.05

CLP causes a systemic inflammation response in mice [20]. Increased plasma extravasation caused by increased vascular permeability is one hallmark of this systemic inflammation. Vascular permeability increased in all organs 8 and 24 h following CLP (Fig. 4a–d). Compared with Isolyte®-treated mice, HES 130/0.4 administration caused significantly decreased vascular permeability in the lung after 8 and 24 h (Fig. 4a). In the kidney (Fig. 4b), the liver (Fig. 4c), and brain tissue (Fig. 4d), HES 130/0.4 decreased the vascular permeability significantly after 24 h following CLP compared with Isolyte® treatment. To specifically address the contribution of glycocalyx degradation on the observed HES effect on vascular permeability changes, we performed additional experiments with the heparanase inhibitor *N*-desulfated/re-*N*-acetylated heparin (NAH). NAH coadministration together with HES abolished the vascular permeability decrease exerted by HES alone by 57–86%, depending on the specific organ (Fig. 4e). Interestingly, the administration of NAH alone did not modulate vascular permeability in our CLP model (Additional file 2: Figure S2). These data indicate that glycocalyx degradation does not mediate vascular permeability changes alone, but rather in addition to direct effects on the vascular endothelial cells. During LPS-induced pulmonary inflammation, vascular permeability increased in all organs 8 and 24 h after LPS inhalation, whereas the vascular permeability in saline-treated control animals did not change (Fig. 5a–d). HES 130/0.4 significantly decreased vascular permeability in the lung, liver, kidney, and brain 24 h after LPS inhalation compared with Isolyte®-treated mice (Fig. 5a–d). To investigate whether the observed protective effect of HES is compound-specific or a common property of colloids in general, we performed additional experiments with albumin. Whereas HES significantly reduced vascular permeability in the lung, liver, kidney, and brain at 24 h after CLP induction compared with Isolyte® treatment, this effect was observed in the brain only with albumin (Additional file 3: Figure S3A–D). To further characterize the severity grade of our CLP model, we analyzed different serum markers for organ damage 24 h after CLP induction. CLP induction did not readily increase serum creatinine (Additional file 3: Figure S3E), whereas it induced a modest increase in serum urea (Additional file 3: Figure S3F). Serum glutamate-pyruvate transaminase (GPT) and glutamic oxaloacetic transaminase (GOT) as markers of hepatic cell injury were also modestly increased 24 h after CLP induction. Serum lactate levels in control animals 24 h after the CLP procedure averaged 2.8 mmol/L, indicating a modest degree of circulatory disorder. All mice survived the 24-h observation period; thus, our CLP was targeted at a rather modest severity level. Neither HES 130/0.4 nor albumin significantly altered the increase in serum urea, GPT, or GOT (Additional file 3: Figure S3F–H).

## Discussion

Our data demonstrate that the administration of HES 130/0.4 significantly reduced increased vascular permeability caused by systemic inflammation after CLP and by pulmonary inflammation following LPS inhalation. Treatment with HES 130/0.4 also reduced the plasma levels of syndecan-1, heparanase, hyaluronic acid, and hyaluronidase activity after CLP or LPS inhalation, indicating a protective effect on the integrity of the vascular glycocalyx. By using IVM of the cremaster and the lung, we demonstrated that HES 130/0.4 maintains the integrity of the vascular glycocalyx during systemic inflammation following CLP and during pulmonary inflammation following LPS inhalation.

The glycocalyx is a delicate structure, and current research has indicated that it is especially prone to degradation during pathological conditions [12]. It has been shown that activation of Toll-like receptor (TLR) 2 or 4 contributes to glycocalyx degradation [31]. The same TLRs are also binding receptors for LPS originating from cell walls of invading bacteria during infections, which was mimicked by CLP or LPS inhalation in this study. In particular, degradation of the endothelial glycocalyx is induced during pulmonary inflammation [32]. Interestingly, by using atomic force microscopy of the compromised lung endothelial glycocalyx, it could be demonstrated that increasing concentrations of HES are capable of modulating the biomechanical properties of the glycocalyx by increasing its thickness and “softness” [33]. This is of interest because it suggests that HES not only may prevent the degradation of the glycocalyx as suggested by the data in this study but also may contribute to its repair.

We showed in this study that the administration of HES attenuates the release of the cleavage enzymes heparanase and hyaluronidase and lowers the shedding of syndecan-1 and hyaluronic acid. To date, it remains unknown how the administration of HES modulates these enzymes. However, heparanase and hyaluronidase are also expressed and released by activated platelets [34, 35]. Platelets are actively involved in the pathogenesis of inflammatory processes and can attach to inflamed endothelial cells [36, 37]. Here, the surface expression of these enzymes on activated platelets was shown to contribute to degradation of the endothelial glycocalyx and the subendothelial extracellular matrix [38]. Hydroxyethyl starches have been shown to influence hemostasis, possibly by scaffolding von Willebrand factor, one of the most important binding receptors for platelets, as well as by directly modulating platelet activation [39]. Although direct experimental evidence is lacking to date, it might be speculated that HES inhibits platelet activation and adhesion to inflamed endothelial cells and thus limits glycocalyx degradation. This would also be in accordance with the results of our previous study where we showed that HES inhibits the formation of platelet-neutrophil aggregates, which also relies on the proper function of platelet surface receptors [40]. In this study, we demonstrated that the administration of HES 130/0.4 modulates leukocyte recruitment during inflammatory processes in vivo [40]. Because the process of glycocalyx shedding on inflamed endothelial cells also enables more efficient interactions of adhesion molecules on leukocytes with its receptors on the inflamed endothelium, the protective effect of HES on glycocalyx integrity in this study could also in part contribute to the effects of HES on leukocyte recruitment.

Leukocyte recruitment is a crucial component of a functional immune response. Thus, in immunocompromised patients, it might be dangerous to abrogate leukocyte function. Indeed, it was shown that the glycocalyx specifically affects leukocyte-endothelial cell interactions, but the pathophysiological relevance has to be further investigated in vivo [41–45]. Regarding HES 130/0.4, our group previously showed that HES administration indeed caused decreased leukocyte adhesion and transmigration in a CLP model [40]. On one hand, in the critically ill patient, systemic inflammatory response syndrome and sepsis are complications that may be associated with an overshooting immune response, which may result in excessive tissue injury, edema formation, and ultimately multiorgan failure. On the other hand, deterioration of leukocyte recruitment could also prevent bacterial clearance of the septic focus. Thus, modulating an inappropriate immune response could be beneficial in some cases, yet detrimental in other situations where bacterial clearance may be the main goal.

The degradation of the endothelial glycocalyx precedes the increase in vascular endothelial permeability during vascular inflammation [15]. We showed that the administration of HES decreases vascular permeability during systemic and pulmonary inflammation. This is in line with previous reports of the effect of HES 130/0.4 on lung edema formation in pigs [46]. In another study, the administration of HES ameliorated the pulmonary vascular permeability disturbances in intensive care patients with sepsis-related acute respiratory distress syndrome [47]. Although the existing preclinical data on glycocalyx shedding during systemic inflammation appear very promising, no human clinical trial has demonstrated that targeting glycocalyx shedding (e.g., by administration of hydrocortisone) translates into a measurable patient benefit in terms of major outcomes [48]. In contrast to previous reports indicating a protective role of NAH administration for glycocalyx shedding following polymicrobial sepsis, to our surprise, we did not observe a protective effect of NAH. Different protocols (e.g. duration of CLP, intensity of resulting systemic inflammation, time points of sample collection following NAH administration and investigated endpoints) might affect the observed effects. Also, tissue differences regarding effects of heparanase inhibition on vascular permeability have to be taken into account [28, 49]. The molecular mechanism of how HES prevents an increase in vascular permeability during inflammation is unknown. A possible explanation could be that HES also modulates neutrophil recruitment into organs during systemic inflammation, a process that enforces local inflammation and boosts the release of vasoactive mediators (e.g., also from participating platelets) [40]. However, it was not the focus of this study to unravel the molecular mechanism of how HES prevents an increase in vascular permeability during inflammation. Further studies are needed to address this point.

We did observe differences in the overall strength of the glycocalyx integrity marker release between CLP and LPS inhalation. CLP is a rather strong stimulus that produces pronounced systemic inflammation resulting in strong alterations in systemic glycocalyx shedding, release of inflammatory mediators, and vascular permeability changes. In contrast, the application of nebulized LPS represents a more localized inflammatory stimulus evoking a more discrete systemic response. This may explain the observed differences with respect to the overall inflammatory effect between the CLP and LPS inhalation. Yet, our data also indicate the rather modest severity of our CLP model. Thus, the findings of this study have to be interpreted bearing in mind that the systemic inflammation present during septic shock in patients may be much more severe than in our animal CLP model, which did not increase mortality in the first 24 h after induction.

However, great caution must be taken when performing volume resuscitation because it is also known that excessive HES 130/0.4 administration leading to hypervolemia may also have deleterious effects on the glycocalyx [50]. In this case, volume overload may trigger the release of the atrial natriuretic peptide, which is known to mediate glycocalyx shedding [51, 52]. Yet, the exact degree to which volume overload, particularly with colloids, may contribute to glycocalyx shedding is still a matter of debate [53]. However, it is noteworthy that this effect seems not to be restricted to colloids but is also a consequence of volume overload. Of note, the administration and dosing regimen of HES and Isolyte® may yield changes in global hemodynamic parameters between the different groups. In our previous study, we performed additional control experiments for the measurement of global hemodynamic variables after CLP induction and regarding the effects of HES administration using the same HES administration regimen and CLP method [40]. Here, no significant differences were found between the control and intervention groups.

Since reports of increased frequencies of renal replacement therapy associated with the use of colloids in critically ill patients, the effect of HES on kidney function has been of special interest [11, 54]. In fact, HES 130/0.4 was banned from the treatment of patients with sepsis. Yet, the underlying mechanisms of HES administration remain elusive. In fact, a variety of HES formulations have been used (e.g., different concentrations, different compounds such as potato or corn starch). Although there have been previous reports pointing to possible interactions of colloids and the glycocalyx, we were still quite surprised to observe a protective effect even in septic animals. The data on the possible beneficial effects of HES presented in this study may potentially be of interest for clinicians and concern patient subpopulations, which have not been addressed yet by the large trials on the topic (VISEP, 6S, CHEST). Yet, owing to the lack of hard outcome parameters in this study (e.g., mortality), future clinical trials are certainly needed to address this point. However, the increased frequency of renal replacement therapy associated with the use of colloids has been shown only for fluid resuscitation in critically ill patients with sepsis, and it could not be demonstrated for perioperative or trauma patients [55, 56]. Causative approaches to explaining these findings remain scarce. Lately, acetate as a common component of available HES formulations has been shown to act as a proinflammatory factor during systemic inflammation in rats [57]. Furthermore, a recent study showed that high dosages (50 ml/kg) of HES 130/0.4 cause deterioration of kidney function in otherwise healthy rats [58]. Yet, the exact effects of hydroxyethyl starches of different compositions on renal function and the development of acute kidney injury remain unclear and clearly warrant further research.

## Conclusions

This study demonstrates that 6% HES 130/0.4 exerts protective effects on glycocalyx integrity during systemic inflammation. This was reflected by lower serum levels of glycocalyx-degrading enzymes, glycocalyx shedding products, and preserved glycocalyx thickness after HES administration in vivo. Furthermore, HES attenuates the increase of vascular permeability during systemic inflammation. Further studies are needed to address the underlying molecular mechanisms.

## Additional files

Additional file 1: **Figure S1.** Effects of HES 130/0.4 on hyaluronidase plasma levels during systemic and pulmonary inflammation. (A) Wild-type mice underwent sham or CLP operation (*n* = 8). Sixty minutes after the procedure, mice received 20 ml/kg Isolyte® (Iso) or HES 130/0.4 (HES) as an infusion over 1 h. The hyaluronidase plasma levels were analyzed 4, 8, and 24 h after the operation. (B) Wild-type mice were exposed to nebulized saline or LPS (*n* = 8). Sixty minutes after the procedure, mice received 20 ml/kg Isolyte® (Iso) or HES 130/0.4 (HES) as an infusion over 1 h. The hyaluronidase plasma levels were analyzed 4, 8, and 24 h after the procedure. Mean ± SEM. * *p* < 0.05. (PDF 606 kb)

Additional file 2: **Figure S2.** Effects of NAH on vascular permeability during systemic inflammation. Wild-type mice underwent a sham or CLP operation (*n* = 4). Twenty-four hours after the operation, the vascular permeability in the lung, liver, kidney, and brain was measured by photometry using the extravasation of Evans blue technique. Mean ± SEM. (PDF 622 kb)

Additional file 3: **Figure S3.** Albumin’s effects on vascular permeability during systemic inflammation and markers of organ tissue damage. Wild-type mice underwent a sham or CLP operation (*n* = 4–7). Sixty minutes after the procedure, mice received 20 ml/kg Isolyte® (Iso), HES 130/0.4 (HES), or albumin 20% as an infusion over 1 h. At 24 h after the operation, vascular permeability in the (**A**) lung, (**B**) liver, (**C**) kidney, and (**D**) brain was measured by photometry using the extravasation of Evans blue technique. Serum creatinine (**E**), urea (**F**), GPT (**G**), and GOT (**H**) were measured 24 h after CLP induction. Mean ± SEM. * *p* < 0.05. (PDF 493 kb)

## Abbreviations

CLP: Cecal ligation and puncture
EB: Evans blue
ELISA: Enzyme-linked immunosorbent assay FITC, Fluorescein isothiocyanate
GOT: Glutamic oxaloacetic transaminase
GPT: Glutamate-pyruvate transaminase
HES: Hydroxyethyl starch
Iso: Isolyte®
IVM: Intravital microscopy
LPS: Lipopolysaccharide
NAH: *N*-desulfated/re-*N*-acetylated heparin
TLR: Toll-like receptor
Vol: Volulyte®

## References

[1] Angus DC, Linde-Zwirble WT, Lidicker J, Clermont G, Carcillo J, Pinsky MR (2001). Epidemiology of severe sepsis in the United States: analysis of incidence, outcome, and associated costs of care. Crit Care Med.

[2] Wilhelms SB, Huss FR, Granath G, Sjoberg F (2010). Assessment of incidence of severe sepsis in Sweden using different ways of abstracting International Classification of Diseases codes: difficulties with methods and interpretation of results. Crit Care Med.

[3] Rossaint J, Zarbock A (2015). Pathogenesis of multiple organ failure in sepsis. Crit Rev Immunol.

[4] Groeneveld AB, Teule GJ, Bronsveld W, van den Bos GC, Thijs LG (1987). Increased systemic microvascular albumin flux in septic shock. Intensive Care Med.

[5] Christ F, Gamble J, Gartside IB, Kox WJ (1998). Increased microvascular water permeability in patients with septic shock, assessed with venous congestion plethysmography (VCP). Intensive Care Med.

[6] Kreimeier U (2000). Pathophysiology of fluid imbalance. Crit Care.

[7] Hahn RG (2014). Why crystalloids will do the job in the operating room. Anaesthesiol Intensive Ther.

[8] Kruer RM, Ensor CR (2012). Colloids in the intensive care unit. Am J Health Syst Pharm.

[9] Bassingthwaighte JB (2006). A practical extension of hydrodynamic theory of porous transport for hydrophilic solutes. Microcirculation.

[10] Mohamed EI, Bayoumi AM (2010). Modeling combined transport of water and charged graded-size molecules across the glomerular capillary wall. Biochem Biophys Res Commun.

[11] Myburgh JA, Finfer S, Bellomo R, Billot L, Cass A, Gattas D, Glass P, Lipman J, Liu B, McArthur C (2012). Hydroxyethyl starch or saline for fluid resuscitation in intensive care. N Engl J Med.

[12] Tarbell JM, Cancel LM (2016). The glycocalyx and its significance in human medicine. J Intern Med.

[13] Reitsma S, Slaaf DW, Vink H, van Zandvoort MA, oude Egbrink MG (2007). The endothelial glycocalyx: composition, functions, and visualization. Pflugers Arch.

[14] Lipowsky HH (2012). The endothelial glycocalyx as a barrier to leukocyte adhesion and its mediation by extracellular proteases. Ann Biomed Eng.

[15] Chelazzi C, Villa G, Mancinelli P, De Gaudio AR, Adembri C (2015). Glycocalyx and sepsis-induced alterations in vascular permeability. Crit Care.

[16] Ushiyama A, Kataoka H, Iijima T (2016). Glycocalyx and its involvement in clinical pathophysiologies. J Intensive Care.

[17] Huang X, Kong G, Li Y, Zhu W, Xu H, Zhang X, Li J, Wang L, Zhang Z, Wu Y (2016). Decitabine and 5-azacitidine both alleviate LPS induced ARDS through anti-inflammatory/antioxidant activity and protection of glycocalyx and inhibition of MAPK pathways in mice. Biomed Pharmacother.

[18] Han S, Lee SJ, Kim KE, Lee HS, Oh N, Park I, Ko E, Oh SJ, Lee YS, Kim D (2016). Amelioration of sepsis by TIE2 activation-induced vascular protection. Sci Transl Med.

[19] Nordling S, Hong J, Fromell K, Edin F, Brannstrom J, Larsson R, Nilsson B, Magnusson PU (2015). Vascular repair utilising immobilised heparin conjugate for protection against early activation of inflammation and coagulation. Thromb Haemost.

[20] Rittirsch D, Huber-Lang MS, Flierl MA, Ward PA (2009). Immunodesign of experimental sepsis by cecal ligation and puncture. Nat Protoc.

[21] Reutershan J, Basit A, Galkina EV, Ley K (2005). Sequential recruitment of neutrophils into lung and bronchoalveolar lavage fluid in LPS-induced acute lung injury. Am J Physiol Lung Cell Mol Physiol.

[22] Zarbock A, Distasi MR, Smith E, Sanders JM, Kronke G, Harry BL, von Vietinghoff S, Buscher K, Nadler JL, Ley K (2009). Improved survival and reduced vascular permeability by eliminating or blocking 12/15-lipoxygenase in mouse models of acute lung injury (ALI). J Immunol.

[23] Radu M, Chernoff J (2013). An in vivo assay to test blood vessel permeability. J Vis Exp.

[24] Block H, Herter JM, Rossaint J, Stadtmann A, Kliche S, Lowell CA, Zarbock A (2012). Crucial role of SLP-76 and ADAP for neutrophil recruitment in mouse kidney ischemia-reperfusion injury. J Exp Med.

[25] Herter JM, Rossaint J, Block H, Welch H, Zarbock A (2013). Integrin activation by P-Rex1 is required for selectin-mediated slow leukocyte rolling and intravascular crawling. Blood.

[26] Zarbock A, Lowell CA, Ley K (2007). Spleen tyrosine kinase Syk is necessary for E-selectin-induced α_L_β_2_ integrin-mediated rolling on intercellular adhesion molecule-1. Immunity.

[27] Rossaint J, Herter JM, Van Aken H, Napirei M, Doring Y, Weber C, Soehnlein O, Zarbock A (2014). Synchronized integrin engagement and chemokine activation is crucial in neutrophil extracellular trap-mediated sterile inflammation. Blood.

[28] Schmidt EP, Yang Y, Janssen WJ, Gandjeva A, Perez MJ, Barthel L, Zemans RL, Bowman JC, Koyanagi DE, Yunt ZX (2012). The pulmonary endothelial glycocalyx regulates neutrophil adhesion and lung injury during experimental sepsis. Nat Med.

[29] Vink H, Duling BR (1996). Identification of distinct luminal domains for macromolecules, erythrocytes, and leukocytes within mammalian capillaries. Circ Res.

[30] Marechal X, Favory R, Joulin O, Montaigne D, Hassoun S, Decoster B, Zerimech F, Neviere R (2008). Endothelial glycocalyx damage during endotoxemia coincides with microcirculatory dysfunction and vascular oxidative stress. Shock.

[31] Pahwa R, Nallasamy P, Jialal I (2016). Toll-like receptors 2 and 4 mediate hyperglycemia induced macrovascular aortic endothelial cell inflammation and perturbation of the endothelial glycocalyx. J Diabetes Complications.

[32] Brettner F, von Dossow V, Chappell D (2017). The endothelial glycocalyx and perioperative lung injury. Curr Opin Anaesthesiol.

[33] Job KM, O’Callaghan R, Hlady V, Barabanova A, Dull RO (2016). The biomechanical effects of resuscitation colloids on the compromised lung endothelial glycocalyx. Anesth Analg.

[34] Vlodavsky I, Eldor A, Haimovitz-Friedman A, Matzner Y, Ishai-Michaeli R, Lider O, Naparstek Y, Cohen IR, Fuks Z (1992). Expression of heparanase by platelets and circulating cells of the immune system: possible involvement in diapedesis and extravasation. Invasion Metastasis.

[35] de la Motte C, Nigro J, Vasanji A, Rho H, Kessler S, Bandyopadhyay S, Danese S, Fiocchi C, Stern R (2009). Platelet-derived hyaluronidase 2 cleaves hyaluronan into fragments that trigger monocyte-mediated production of proinflammatory cytokines. Am J Pathol.

[36] Rossaint J, Zarbock A (2015). Platelets in leucocyte recruitment and function. Cardiovasc Res.

[37] Herter JM, Rossaint J, Zarbock A (2014). Platelets in inflammation and immunity. J Thromb Haemost.

[38] Albeiroti S, Ayasoufi K, Hill DR, Shen B, de la Motte CA (2015). Platelet hyaluronidase-2: an enzyme that translocates to the surface upon activation to function in extracellular matrix degradation. Blood.

[39] Levi M, Jonge E (2007). Clinical relevance of the effects of plasma expanders on coagulation. Semin Thromb Hemost.

[40] Rossaint J, Berger C, Kraft F, Van Aken H, Giesbrecht N, Zarbock A (2015). Hydroxyethyl starch 130/0.4 decreases inflammation, neutrophil recruitment, and neutrophil extracellular trap formation. Br J Anaesth.

[41] Petri B, Phillipson M, Kubes P (2008). The physiology of leukocyte recruitment: an in vivo perspective. J Immunol.

[42] Mulivor AW, Lipowsky HH (2002). Role of glycocalyx in leukocyte-endothelial cell adhesion. Am J Physiol Heart Circ Physiol.

[43] Constantinescu AA, Vink H, Spaan JA (2003). Endothelial cell glycocalyx modulates immobilization of leukocytes at the endothelial surface. Arterioscler Thromb Vasc Biol.

[44] Chappell D, Heindl B, Jacob M, Annecke T, Chen C, Rehm M, Conzen P, Becker BF (2011). Sevoflurane reduces leukocyte and platelet adhesion after ischemia-reperfusion by protecting the endothelial glycocalyx. Anesthesiology.

[45] McDonald KK, Cooper S, Danielzak L, Leask RL (2016). Glycocalyx degradation induces a proinflammatory phenotype and increased leukocyte adhesion in cultured endothelial cells under flow. PLoS One.

[46] Balkamou X, Xanthos T, Stroumpoulis K, Moutzouris DA, Rokas G, Agrogiannis G, Demestiha T, Patsouris E, Papadimitriou L (2010). Hydroxyethyl starch 6% (130/0.4) ameliorates acute lung injury in swine hemorrhagic shock. Anesthesiology.

[47] Huang CC, Kao KC, Hsu KH, Ko HW, Li LF, Hsieh MJ, Tsai YH (2009). Effects of hydroxyethyl starch resuscitation on extravascular lung water and pulmonary permeability in sepsis-related acute respiratory distress syndrome. Crit Care Med.

[48] Keh D, Trips E, Marx G, Wirtz SP, Abduljawwad E, Bercker S, Bogatsch H, Briegel J, Engel C, Gerlach H (2016). Effect of hydrocortisone on development of shock among patients with severe sepsis: the HYPRESS randomized clinical trial. JAMA.

[49] Lygizos MI, Yang Y, Altmann CJ, Okamura K, Hernando AA, Perez MJ, Smith LP, Koyanagi DE, Gandjeva A, Bhargava R (2013). Heparanase mediates renal dysfunction during early sepsis in mice. Physiol Rep.

[50] Chappell D, Bruegger D, Potzel J, Jacob M, Brettner F, Vogeser M, Conzen P, Becker BF, Rehm M (2014). Hypervolemia increases release of atrial natriuretic peptide and shedding of the endothelial glycocalyx. Crit Care.

[51] Bruegger D, Jacob M, Rehm M, Loetsch M, Welsch U, Conzen P, Becker BF (2005). Atrial natriuretic peptide induces shedding of endothelial glycocalyx in coronary vascular bed of guinea pig hearts. Am J Physiol Heart Circ Physiol.

[52] Bruegger D, Schwartz L, Chappell D, Jacob M, Rehm M, Vogeser M, Christ F, Reichart B, Becker BF (2011). Release of atrial natriuretic peptide precedes shedding of the endothelial glycocalyx equally in patients undergoing on- and off-pump coronary artery bypass surgery. Basic Res Cardiol.

[53] Hahn RG (2015). Must hypervolaemia be avoided? A critique of the evidence. Anaesthesiol Intensive Ther.

[54] Bagshaw SM, Chawla LS (2013). Hydroxyethyl starch for fluid resuscitation in critically ill patients. Can J Anaesth.

[55] Gillies MA, Habicher M, Jhanji S, Sander M, Mythen M, Hamilton M, Pearse RM (2014). Incidence of postoperative death and acute kidney injury associated with i.v. 6% hydroxyethyl starch use: systematic review and meta-analysis. Br J Anaesth.

[56] Leberle R, Ernstberger A, Loibl M, Merkl J, Bunz M, Creutzenberg M, Trabold B (2015). Association of high volumes of hydroxyethyl starch with acute kidney injury in elderly trauma patients. Injury.

[57] Schimmer RC, Urner M, Voigtsberger S, Booy C, Roth Z’Graggen B, Beck-Schimmer B, Schlapfer M (2016). Inflammatory kidney and liver tissue response to different hydroxyethylstarch (HES) preparations in a rat model of early sepsis. PLoS One.

[58] Schick MA, Baar W, Bruno RR, Wollborn J, Held C, Schneider R, Flemming S, Schlegel N, Roewer N, Neuhaus W (2015). Balanced hydroxyethylstarch (HES 130/0.4) impairs kidney function in-vivo without inflammation. PLoS One.

