# Supplementary figures and images for: 6% Hydroxyethyl starch (HES 130/0.4) diminishes glycocalyx degradation and decreases vascular permeability during systemic and pulmonary inflammation in mice
Source: Crit Care. 2018 May 1;22:111. doi: 10.1186/s13054-017-1846-3 (PMC5930811; doi:10.1186/s13054-017-1846-3)

Figure S1

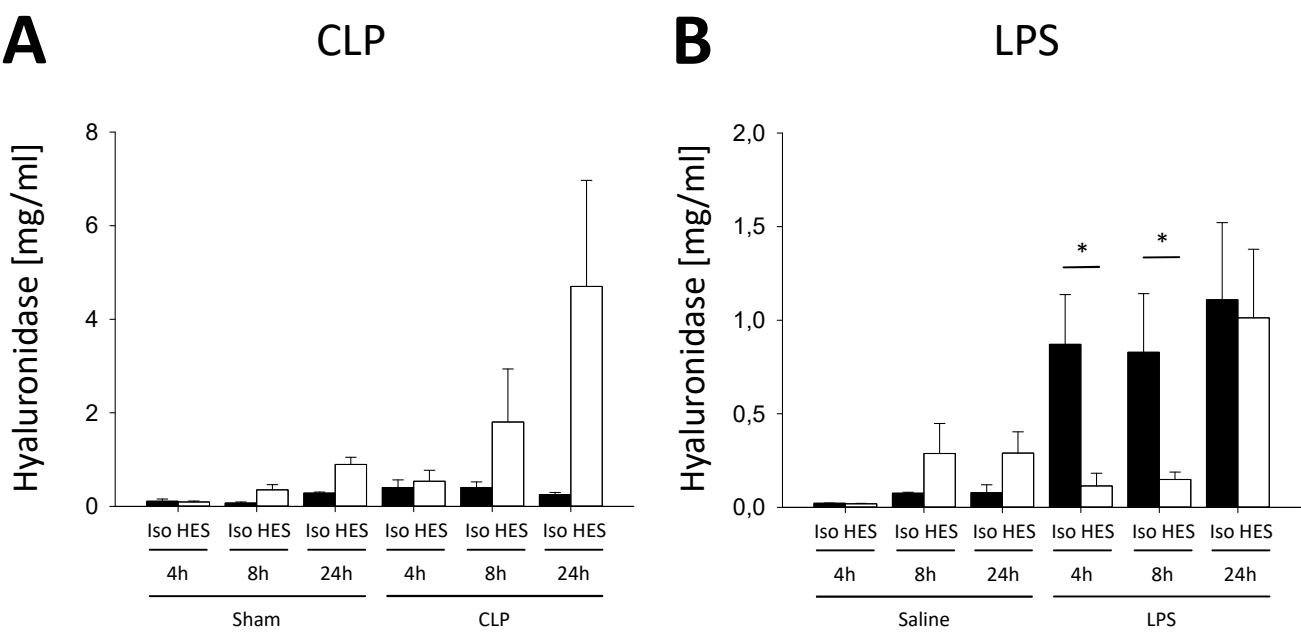

Supplement: Supplementary file 1 — Figure S1. Effects of HES 130/0.4 on hyaluronidase plasma levels during systemic and pulmonary inflammation. (A) Wild-type mice underwent sham or CLP operation (n = 8). Sixty minutes after the procedure, mice received 20 ml/kg Isolyte® (Iso) or HES 130/0.4 (HES) as an infusion over 1 h. The hyaluronidase plasma levels were analyzed 4, 8, and 24 h after the operation. (B) Wild-type mice were exposed to nebulized saline or LPS (n = 8). Sixty minutes after the procedure, mice received 20 ml/kg Isolyte® (Iso) or HES 130/0.4 (HES) as an infusion over 1 h. The hyaluronidase plasma levels were analyzed 4, 8, and 24 h after the procedure. Mean ± SEM. * p < 0.05. (PDF 606 kb) [file 13054_2017_1846_MOESM1_ESM.pdf]

# Figure S2

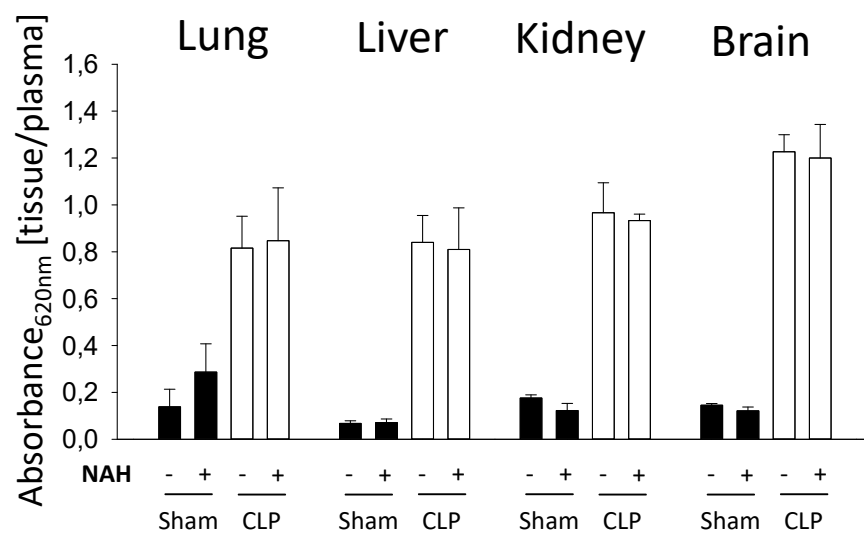

Supplement: Supplementary file 2 — Figure S2. Effects of NAH on vascular permeability during systemic inflammation. Wild-type mice underwent a sham or CLP operation (n = 4). Twenty-four hours after the operation, the vascular permeability in the lung, liver, kidney, and brain was measured by photometry using the extravasation of Evans blue technique. Mean ± SEM. (PDF 622 kb) [file 13054_2017_1846_MOESM2_ESM.pdf]

# Figure S3

**A**

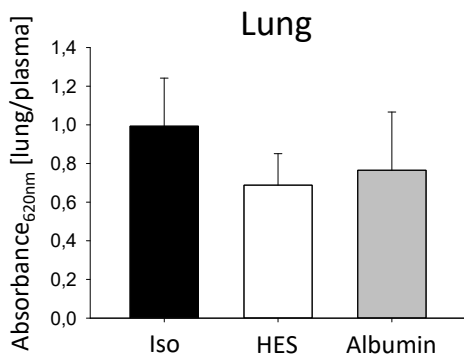

**B**

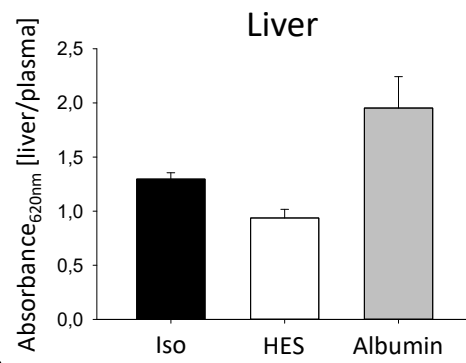

**C**

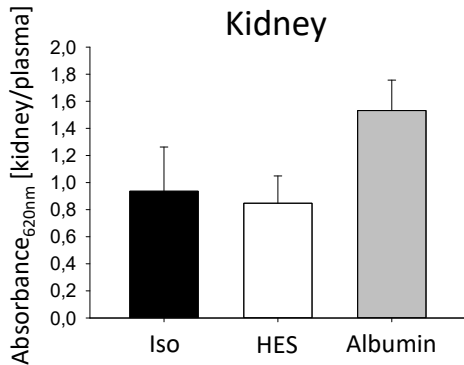

**D**

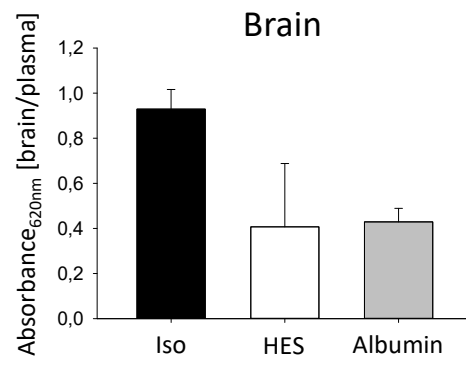

**E**

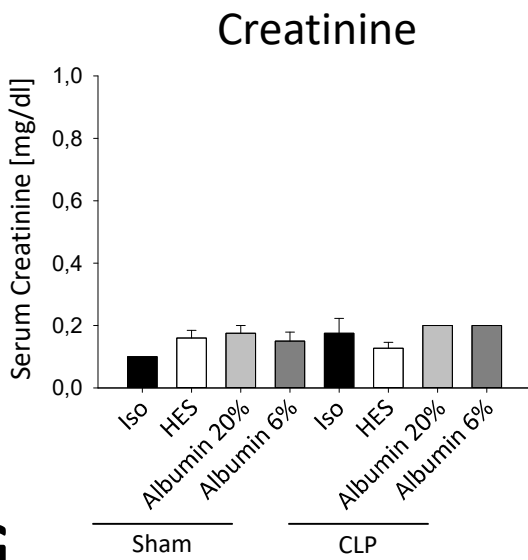

**F**

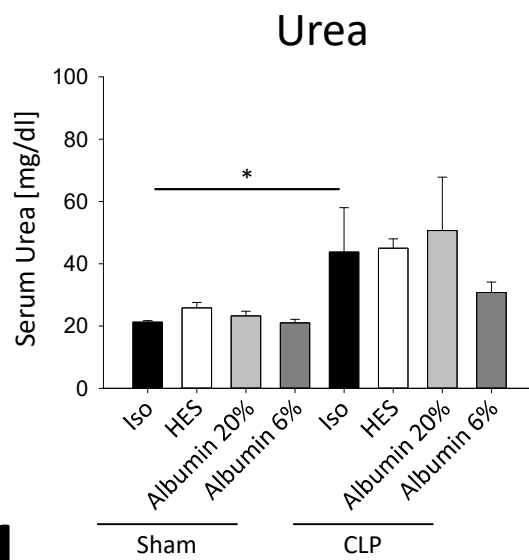

**G**

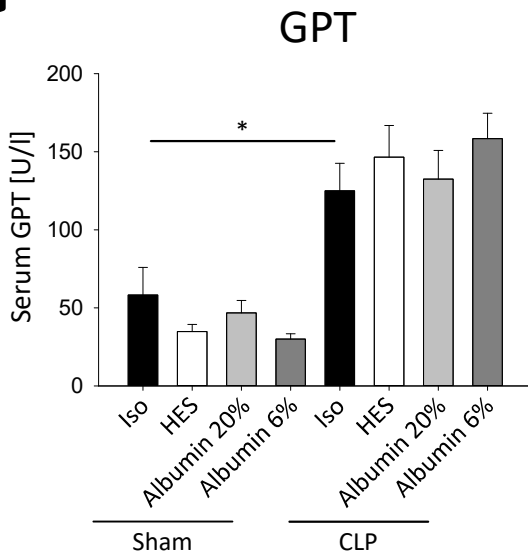

**H**

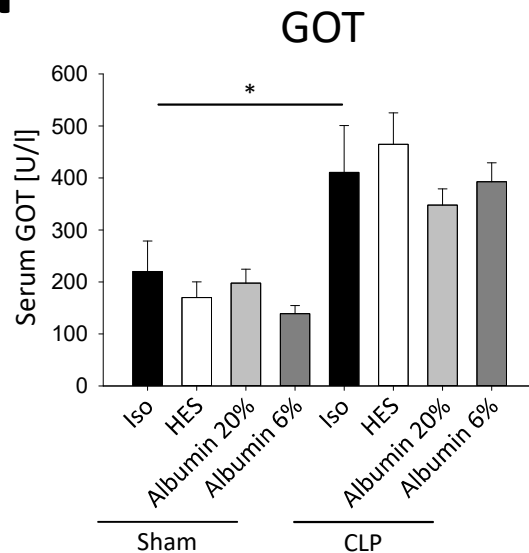

Supplement: Supplementary file 3 — Figure S3. Albumin’s effects on vascular permeability during systemic inflammation and markers of organ tissue damage. Wild-type mice underwent a sham or CLP operation (n = 4–7). Sixty minutes after the procedure, mice received 20 ml/kg Isolyte® (Iso), HES 130/0.4 (HES), or albumin 20% as an infusion over 1 h. At 24 h after the operation, vascular permeability in the (A) lung, (B) liver, (C) kidney, and (D) brain was measured by photometry using the extravasation of Evans blue technique. Serum creatinine (E), urea (F), GPT (G), and GOT (H) were measured 24 h after CLP induction. Mean ± SEM. * p < 0.05. (PDF 493 kb) [file 13054_2017_1846_MOESM3_ESM.pdf]
